# Supplementary material for: Fetal yawning and mouth openings: Frequency, developmental trends, and association with birth weight
Source: PLoS One. 2026 Feb 25;21(2):e0341339. doi: 10.1371/journal.pone.0341339 (PMC12935229; doi:10.1371/journal.pone.0341339)
Supplement: S4 File — (PDF) [file pone.0341339.s004.pdf]

code

```
yawns <-  glm(SBAD ~ GA_scaled+bw_scaled+sex, offset=log(PER_COD) , data=yawn, family=poisson
, na.action=na.omit)
summary(yawns)
yawns_nosex <-  glm(SBAD ~ GA_scaled+bw_scaled, offset=log(PER_COD) , data=yawn, family=
poisson, na.action=na.omit)
summary(yawns_nosex)

anova(yawns_nosex, yawns)

mos <-  glm(OM ~ GA_scaled+bw_scaled+sex, offset=log(PER_COD) , data=yawn, family=poisson,
na.action=na.omit)
summary(mos)
mos_nosex <-  glm(OM ~ GA_scaled+bw_scaled, offset=log(PER_COD) , data=yawn, family=poisson,
na.action=na.omit)
summary(mos_nosex)

anova(mos_nosex, mos)


yp <-  lm(yawnperc ~ GA_scaled+PESO_NASCITA , data=yawn3, na.action=na.omit)
summary(yp)
```

log

```
#yawning model with sex

Call:
glm(formula = SBAD ~ GA_scaled + bw_scaled + sex, family = poisson,
    data = yawn, na.action = na.omit, offset = log(PER_COD))

Coefficients:
             Estimate Std. Error z value Pr(>|z|)
(Intercept) -14.02913    0.30920 -45.373   <2e-16 ***
GA_scaled     0.03938    0.20853   0.189   0.8502
bw_scaled    -0.41291    0.19194  -2.151   0.0315 *
sex1          0.13305    0.37183   0.358   0.7205
---
Signif. codes:  0 ‘***’ 0.001 ‘**’ 0.01 ‘*’ 0.05 ‘.’ 0.1 ‘ ’ 1

(Dispersion parameter for poisson family taken to be 1)

    Null deviance: 47.557  on 31  degrees of freedom
Residual deviance: 42.319  on 28  degrees of freedom
AIC: 93.302

Number of Fisher Scoring iterations: 5

#yawning model without sex

Call:
```

```
glm(formula = SBAD ~ GA_scaled + bw_scaled, family = poisson,
    data = yawn, na.action = na.omit, offset = log(PER_COD))

Coefficients:
             Estimate Std. Error z value Pr(>|z|)
(Intercept) -13.94513    0.19444 -71.721   <2e-16 ***
GA_scaled     0.04673    0.20729   0.225   0.8216
bw_scaled    -0.41974    0.19149  -2.192   0.0284 *
---
Signif. codes:  0 ‘***’ 0.001 ‘**’ 0.01 ‘*’ 0.05 ‘.’ 0.1 ‘ ’ 1

(Dispersion parameter for poisson family taken to be 1)

    Null deviance: 47.557  on 31  degrees of freedom
Residual deviance: 42.448  on 29  degrees of freedom
AIC: 91.432

Number of Fisher Scoring iterations: 5
#ANOVA (model comparison for yawning models)
Analysis of Deviance Table

Model 1: SBAD ~ GA_scaled + bw_scaled
Model 2: SBAD ~ GA_scaled + bw_scaled + sex
  Resid. Df Resid. Dev Df Deviance Pr(>Chi)
1        29     42.448
2        28     42.319  1  0.12981   0.7186

#mouth openings model with sex

Call:
glm(formula = OM ~ GA_scaled + bw_scaled + sex, family = poisson,
    data = yawn, na.action = na.omit, offset = log(PER_COD))

Coefficients:
             Estimate Std. Error z value Pr(>|z|)
(Intercept) -12.61479    0.15219 -82.890  < 2e-16 ***
GA_scaled    -0.48683    0.10596  -4.594 4.34e-06 ***
bw_scaled    -0.09914    0.08272  -1.198    0.231
sex1          0.19479    0.17805   1.094    0.274
---
Signif. codes:  0 ‘***’ 0.001 ‘**’ 0.01 ‘*’ 0.05 ‘.’ 0.1 ‘ ’ 1

(Dispersion parameter for poisson family taken to be 1)

    Null deviance: 121.160  on 31  degrees of freedom
Residual deviance:  93.285  on 28  degrees of freedom
AIC: 187.27

Number of Fisher Scoring iterations: 5

#mouth openings model without sex
```

```
Call:
glm(formula = OM ~ GA_scaled + bw_scaled, family = poisson, data = yawn,
    na.action = na.omit, offset = log(PER_COD))

Coefficients:
             Estimate Std. Error  z value Pr(>|z|)
(Intercept) -12.48855    0.09438 -132.326  < 2e-16 ***
GA_scaled    -0.47969    0.10598   -4.526 6.01e-06 ***
bw_scaled    -0.10132    0.08320   -1.218    0.223
---
Signif. codes:  0 '***' 0.001 '**' 0.01 '*' 0.05 '.' 0.1 ' ' 1

(Dispersion parameter for poisson family taken to be 1)

    Null deviance: 121.160  on 31  degrees of freedom
Residual deviance:  94.505  on 29  degrees of freedom
AIC: 186.49

Number of Fisher Scoring iterations: 5

#ANOVA (model comparison for mouth openings models)

Analysis of Deviance Table

Model 1: OM ~ GA_scaled + bw_scaled
Model 2: OM ~ GA_scaled + bw_scaled + sex
  Resid. Df Resid. Dev Df Deviance Pr(>Chi)
1        29     94.505
2        28     93.285  1     1.22   0.2694
>
#yawning percentage model

Call:
lm(formula = yawnperc ~ GA_scaled + PESO_NASCITA, data = yawn3,
    na.action = na.omit)

Residuals:
     Min       1Q   Median       3Q      Max
-0.41650 -0.13678  0.00588  0.09536  0.61462

Coefficients:
               Estimate Std. Error t value Pr(>|t|)
(Intercept)   5.477e-01  4.466e-01   1.226   0.2315
GA_scaled     1.629e-01  5.554e-02   2.932   0.0071 **
PESO_NASCITA -9.568e-05  1.363e-04  -0.702   0.4892
---
Signif. codes:  0 '***' 0.001 '**' 0.01 '*' 0.05 '.' 0.1 ' ' 1

Residual standard error: 0.2484 on 25 degrees of freedom
Multiple R-squared:  0.2562,    Adjusted R-squared:  0.1967
```

```
F-statistic: 4.305 on 2 and 25 DF,  p-value: 0.02473
```
