# Supplementary material for: Fetal yawning and mouth openings: Frequency, developmental trends, and association with birth weight
Source: PLoS One. 2026 Feb 25;21(2):e0341339. doi: 10.1371/journal.pone.0341339 (PMC12935229; doi:10.1371/journal.pone.0341339)
Supplement: Attachment — Submitted filename: REV_PlosOne.docx [file pone.0341339.s005.docx]

**General comment**

Overall, this is a very interesting and carefully conducted study, even if it may not be entirely groundbreaking. The topic of fetal yawning is engaging, and the authors’ attention to methodological detail is commendable. That said, the introduction feels quite long and dense in places. Personally, I find that presenting the hypotheses separately from the main flow of the introduction can interrupt the narrative, and a more integrated approach might help the reader follow the reasoning from theory to research questions more smoothly.

The discussion provides a clear summary of the findings, but it could be made more engaging. At times, the results are presented without extensive connection to existing literature, which makes it harder to fully appreciate their relevance. Additionally, ending the discussion by emphasizing study limitations tends to overshadow the contributions of the work. Highlighting the study’s strengths and the insights it provides, while still acknowledging limitations in a balanced and modest way, could help the discussion leave a stronger impression.

**TITLE** - The title is clear and informative, giving a good sense of the main focus of the study. It accurately reflects the variables examined, fetal yawning, non-yawning mouth openings, and birth weight, as well as the key aspects investigated, such as frequency and stability. One minor point to consider is that it reads a bit long and technical; simplifying it slightly or making it more fluid could make it even more accessible to a broader readership, without losing specificity.

**Introduction**
The introduction provides a very thorough overview of the main theories on yawning and does a good job of integrating both phylogenetic and ontogenetic perspectives. At the same time, it feels quite dense and occasionally repetitive, with many citations clustered together. Some sections lean more toward summarizing the literature than synthesizing it into a coherent argument leading to the rationale of the study. It might be helpful to clarify more explicitly why fetal yawning deserves specific attention beyond the “blind spot” of the brain cooling hypothesis.

Some statements could benefit from slightly more cautious wording. For example, the text sometimes presents the brain cooling hypothesis as a well-established or “unifying” explanation, when it is still a topic of active debate. A softer tone here would better reflect the ongoing discussion in the field. Additionally, the transition from broad theories about yawning in adults to observations in fetuses could be made smoother, to help the reader understand the link between macro-level and micro-level perspectives.

In the section on modulatory factors (lines 45–55), the numerous examples listed (stress, hunger, pain, arousal, thermoregulation) could be more clearly distinguished between well-supported empirical findings and more speculative associations, to avoid overinflating claims.

The discussion of the brain cooling hypothesis (lines 60–79) is very thorough, but could benefit from a more balanced approach. At present, it emphasizes supporting evidence while downplaying limitations, such as the difficulty of measuring brain temperature changes in non-rodent species. Phrases like “unparalleled explanatory and predictive power” might be softened to present the strengths of the hypothesis without overstating them.

The section on contagious yawning (lines 89–101) is relevant, but it could more clearly separate proximate and ultimate explanations. While the Attentional Bias and Emotional Bias hypotheses are mentioned, their connection to evolutionary theory or the broader framework of yawning could be more explicitly clarified. A reference to recent work on zebrafish could also strengthen this section.

The discussion of yawning in poikilotherms (lines 102–111) adds an interesting phylogenetic perspective. Some of the interpretations, such as suggesting that yawning in fish reflects “primitive functions,” could be presented more tentatively, acknowledging that this remains a hypothesis rather than established fact.

The section on fetal yawning (lines 112–124) introduces the core topic well, but the logical flow could be clearer. Presenting Walusinski’s critique followed immediately by Gallup & Eldakar’s counterpoint leaves the reader uncertain about what is empirically known. Phrasing like “does not falsify the brain cooling hypothesis” could be reframed more positively as an opportunity to refine and extend the theory.

The discussion of empirical variability (lines 125–155) is one of the stronger parts of the introduction, as it addresses methodological inconsistencies in prior studies. However, this comes relatively late in the text, after a substantial theoretical discussion. Bringing these points earlier could help the reader understand why the present study is needed. Additionally, the critique of Reissland et al. could be softened by acknowledging their pioneering role, even while highlighting the methodological improvements of later studies.

**Methods**

The sample size and gestational age range (n = 32 fetuses, 23–31 weeks) are reasonable given practical constraints, but it is worth noting that the study may have limited power to detect subtle developmental trends, which should be considered when interpreting hypotheses H2–H4.

**Discussion**
The discussion clearly follows the results but could benefit from a more engaging narrative. At present, it mostly restates findings without fully connecting them to broader theoretical perspectives, such as the brain cooling hypothesis or the evolutionary and developmental context of yawning. Interpretations, for example regarding the negative association between yawning frequency and birth weight, are thoughtfully proposed but remain somewhat ambiguous. It might help to guide the reader toward which explanations are more plausible, while maintaining appropriate caution.

The focus on methodological validity is commendable, but the discussion could further highlight the study’s contribution. Ending with limitations, while important, may leave the reader without a clear sense of the significance of the findings. Expanding briefly on implications for fetal neurobehavioral assessment or the study of yawning modulation across species could strengthen the impact.

Overall, the study appears methodologically sound and makes an interesting contribution, but the discussion would be enhanced by a tighter integration of results with theory, clearer prioritization of interpretations, and a more conclusive closing statement that emphasizes what the study adds to the field.
