# Supplementary material for: Fetal yawning and mouth openings: Frequency, developmental trends, and association with birth weight
Source: PLoS One. 2026 Feb 25;21(2):e0341339. doi: 10.1371/journal.pone.0341339 (PMC12935229; doi:10.1371/journal.pone.0341339)
Supplement: Attachment — Submitted filename: rebuttal_auresp_2.docx [file pone.0341339.s008.docx]

**Response to Academic Editor and Reviewer**

**Academic** **Editor’s comments**

Q1: In my evaluation of the revised manuscript, I noticed an error in reference to the existing literature that should be addressed. On page 4, lines 73-75, it is stated that brain temperature modulation following yawning has only been documented in rats when it has been reported in at least two other species as well. The first is budgerigars (Melopsittacus undulatus), and the second, most recently, is mice. I have provided these references below, which should be included for accuracy in the final version.

Gallup, A. C., Herron, E., Militello, J., Swartwood, L., Cortes, C., & Eguibar, J. R. (2017). Thermal imaging reveals sizable shifts in facial temperature surrounding yawning in budgerigars (Melopsittacus undulatus). *Temperature*, *4*(4), 429-435.

Alam, M. T., Ahasan, M. M., Shimizu, S., Murata, Y., Taniguchi, M., & Yamaguchi, M. (2025). Differential potentiation of odor aversion and yawning by melanocortin 4 receptor signaling in distinct regions of the ventral striatum. *Frontiers in Neuroscience*, *19*, 1668410.

R1: We thank the Academic Editor for spotting this oversight. We have included these citations and the corresponding information in the revised introduction (see below):

“It is important to note, however, that evidence of this modulation is currently available only for rats (25), budgerigars (26) and mice (27), and additional studies are needed to confirm these results in other species.”

**Reviewers' comments**

Reviewer #1:

Q1: In reviewing the authors' replies to both reviewers' points, I think the additions and alterations that the authors have made are adequate and reasonable. If anything, the final paragraph (changed in answer to Rev 2) might be a little too effusive or "expansive" on the yawning conclusions, e.g. "compel a reevaluation of the theoretical functions of yawning". I might have phrased this as "compel continuing evaluation of the theorized functions of yawning across ontogeny as well as phylogeny", which has clearly been relatively neglected.
However, one cannot never write for the many different readers from different backgrounds in making fine points.

R1: We agree with the reviewer’s assessment and very much appreciate her suggestion for an alternative, more balanced phrasing, which we have adopted in the revised version (see below).

“These insights not only open new avenues for the clinical assessment of fetal neurobehavioral status but also compel continuing evaluation of the theorized functions of yawning across ontogeny as well as phylogeny, suggesting the presence in fetuses of a modulatory dynamic closer to extra-uterine life than previously understood.”

Q2: To the mind of this reviewer, the authors have made good use of both reviews and have improved the paper where criticism was given. I will be very interested in follow-up studies where "stress" has a stronger proxy, as well as where the temperature of the uterine environment and the current metabolic output of the fetus might be measured.

R2: We wish to deeply thank the reviewer for the genuinely helpful criticism and support she provided. We hope to be able in the future to carry out studies suited to test more directly the hypothesis of a stress-related yawning modulation in fetuses, or that this study can inspire other scholars to conduct similar studies.

Q3: With respect to editing, there are a few places where grammar fixes could be made. For instance,
a) Ref 1 has two "." at end of title and the species names are not italicized or underlined. (Maybe they are not in original title...I forget.)
b) Repetitive use of "methodological rigor" in ll 380-381
c) l. 371 "nonetheless" is incorrectly used as a conjunction. But mostly it is stylistically fine.

R3: Thanks for these comments, we made the fixes in the revised version. In particular:

a) The title was corrected, removing the dot and italicizing the species names;

b) “Methodologically rigorous study” was replaced with “robust assessment”, in order to avoid the repetition. The sentence now reads “ This methodological rigor, combined with the novel finding of a seeming stress-related modulation in healthy fetuses, makes this arguably the most robust assessment of this phenomenon to date”

c) We have corrected the sentence by replacing the comma with a period and starting a new sentence with "Nonetheless" (now used correctly as a sentence connector rather than a conjunction).

Reviewer #2:

Q1: Thank you for addressing all my comments thoroughly and clearly. The revisions you implemented fully resolve the issues I had raised and substantially improve the manuscript. I have no further remarks and am satisfied with the final version, supporting the acceptance of the paper in its current form.

R1: We wish to thank the reviewer for their constructive criticism and for their positive final evaluation of our work. We are pleased that the revisions have addressed all concerns and that the manuscript is now considered suitable for publication.
